# MCM10 expression is linked to cervical cancer aggressiveness

**DOI:** 10.3389/fmmed.2023.1009903

**Published:** 2023-02-22

**Authors:** Sumayyah M. Q. Ahmed, Suparna Laha, Ranajit Das, Mariam Anjum Ifthikar, Shankar Prasad Das

**Affiliations:** ^1^ Yenepoya Research Centre, Yenepoya (Deemed to be University), Mangalore, India; ^2^ Department of Oncology, Yenepoya Medical College Hospital, Yenepoya (Deemed to be University), Mangalore, India

**Keywords:** cervical cancer, minichromosome maintenance protein 10, DNA replication, origin firing, cancer aggressiveness, qRT-PCR, immunocytochemistry

## Abstract

Cervical cancer screening is a challenge mainly in developing countries. In developed countries, both incidence and mortality rates have been decreasing due to well organized screening programs. One of the potential biomarkers being exploited are the minichromosome maintenance proteins (MCMs), which show both specificity and sensitivity. MCM2-7 are involved in DNA replication initiation and elongation, and the MCM subunits are highly expressed in malignant tissues. Unlike other MCMs, MCM10, which is not part of the core helicase complex, is a critical determinant of origin activation and its levels are limiting in cancer cells. In this study, we performed bioinformatic analysis on the expression profile of all DNA replication associated MCM proteins in cervical cancer. MCM10 showed a relatively higher expression profile compared to the other MCMs. The mRNA expression levels of the MCMs were significantly increased in tumour tissues compared to normal, and MCM10 showed a fold change of 3.4. In order to understand if MCM10 is associated with the aggressiveness of cervical cancer, we looked into the mRNA expression pattern of MCM10 in three cervical cancer cell lines and one normal cervical cell line. MCM10 expression was significantly higher in the case of the more aggressive cancer cell line HeLa compared to controls. MCM10, therefore, can serve as a prominent biomarker for cancer progression and thus aid in early detection to control the spread of cancer cells. Our results show that MCM10 expression levels in cervical cancer cell lines are associated with cancer aggressiveness, demonstrating its clinical significance.

## Introduction

According to the global cancer statistics 2020, Globocan has estimated the worldwide incidence and mortality for 36 cancers in 185 countries. Cervical cancer is the fourth most common cancer in women, accounting for an estimated 60,4127 new cases, with developing countries accounting for more than 85% of newly diagnosed cases (Global Cancer Observatory, 2020). In 2020, cervical cancer was the leading cause of cancer-related death among women in Central America, Sub-Saharan Africa, South-Central Asia, and Melanesia (Arbyn et al., 2020). Due to the lack of early symptoms and detection tools, cervical cancer is mostly diagnosed at advanced stages, which raises an urgent global need to enhance the efficacy and sensitivity of the cytological screening program and look for promising cancer biomarkers that are closely related to tumor development. The discovery of key molecules with abnormal expression patterns has been a significant step for the diagnosis of cervical cancer, which is mostly associated with Human papillomavirus (HPV) infection (Zur Hausen, 2000; Temesgen et al., 2021). More than 200 types of human papillomavirus (HPV) have been identified, among which HPV 16 and HPV 18 cause 70% of cervical cancers and pre-cancerous cervical lesions (Fernandes et al., 2005; Temesgen et al., 2021). HPV infection causes altered expression of HPV E6 and E7 proteins in host cells, which results in increased levels of cell cycle proteins such as p16, Ki67 and Minichromosome Maintenance proteins (Kaur et al., 2019). Despite recent improvements in HPV testing sensitivity, there is still a high percentage of false negative results, part of which accounts for the fact that HPV has been found to be negative in 5.5%–11% of cervical cancers (Xing et al., 2021). So early detection becomes crucial, and hence the search for markers that dictate the aggressiveness of cancer.

As DNA replication is perturbed in cancer cells, the MCM proteins are the most extensively evaluated cell cycle markers in several cancers (Yu et al., 2020). The MCM family is a group of proteins that is required for accurate genome duplication, cell cycle progression, genome stability and disease (Tye, 1994; Jackson et al., 2014). They are used as a screening test for the abnormal expression of human papillomavirus associated with cervical cancer (Griffin et al., 2015; Saritha et al., 2018). In eukaryotes, MCM consists of six gene products (MCM 2–7), which form a heterohexamer, a core component of the replicative helicase complex that unwinds the DNA template during the process of DNA replication (Seo and Kang, 2018). MCM’s play an important role in origin activation and are crucial for origin unwinding, which is a tightly regulated process (Bochman and Schwacha, 2009). They are distributed in a heterogenous fashion across the chromosome, and the amount of loaded MCM corresponds to the replication timing (Das et al., 2015). MCM1 plays an important role in the initiation of DNA replication and is involved in the transcriptional regulation of several cell cycle regulated genes (Chang et al., 2003). MCM8 and the MCM9 complex is involved in homologous recombination-mediated double-strand break repair and also in chromosomal rearrangements (Nishimura et al., 2012). Another member of the MCM family which is not part of the MCM core complex is MCM10. It binds to the CMG complex for the final trigger along with Cdc45(Lõoke et al., 2017; Rios-Morales et al., 2019) during replication initiation and is thus crucial for DNA origin firing (Yeeles et al., 2015; Mayle et al., 2019). There are other MCMs that are involved in proper DNA segregation and play an important role in maintaining genome stability (Mehta et al., 2022). Overexpression of the MCM10 gene has been linked to a number of tumors, including breast, lung, prostate and urothelial carcinoma (Cui et al., 2018; Murayama et al., 2021; Shao et al., 2021; Zhou et al., 2021) and is thus critical for maintaining genome integrity. However, the role of MCM10 in the development of cervical cancer, its link to aggressiveness is unknown. Other studies have shown that MCM10 overexpression promotes cell proliferation (Cui et al., 2018) and MCM10 deficiency causes changes in DNA synthesis that result in defective replisome induced mutagenesis (Becker et al., 2014). In this study, using bioinformatic analysis of oncology databases we assessed the expression of MCMs in major cancers that affect women, including cervical cancer. We highlight the clinical significance of MCM10 expression through experimental validation in cervical cancer cell lines.

## Materials and methods

### Gene expression profiling interactive analysis dataset

A Gene Expression Profiling Interactive Analysis (GEPIA) dataset has been developed for analyzing the RNA sequencing expression data of approximately 9,000 carcinomas and 8,000 normal samples from TCGA and GTEx projects through a standard processing pipeline (http://gepia.cancer-pku.cn/). GEPIA can provide differential expression analysis of cancers that are over-expressed and under-expressed, correlation analysis, gene expression profiles of tumors and normal samples and patient survival analysis. It can also provide the transcript per million of the differentially expressed genes to show relative expression levels. In this study, GEPIA datasets were used to analyze the different expression levels of MCMs in cervical cancer and normal tissues. Using the ANOVA method, the cut-off fold change was set at 1, and a *p*-value < 0.01 was considered significant.

### cBioPortal for cancer genomics

The web resource provides tools for exploring, visualizing and analyzing the multidimensional genomics data for different types of cancer. The portal has customized data storage to explore the genetic alterations across genes and provides graphical summaries of gene-level data from multiple platforms. The genomic data sets can be queried by a single cancer study or by querying multiple cancer studies using the cBioPortal for Cancer Genomics (https://www.cbioportal.org/) (Gao et al., 2013). In the single cancer study, we can explore and visualize the genomic alterations of all the desired genes as an Oncoprint, which is a graphical summary of genomic alteration. Here we have analyzed the cervical squamous cell carcinoma and cervical adenocarcinoma datasets from The Cancer Genome Atlas (TCGA), cBioPortal. The OncoPrint and cancer type summary were obtained from cBioPortal (Cerami et al., 2012).

### UCSC Xena

UCSC Xena is an online exploration tool for public and private, multi-omics, and clinical or phenotype data (http://xena.ucsc.edu/). The data were preprocessed and stored at the UCSC Xena platform’s data hubs, along with many public cancer datasets, allowing researchers to investigate multiple protein expression in a single or multiple cancer types. We have used Xena to compare the TCGA tumor samples to GTEx normal samples for all the MCMs in cervical samples to explore the fold change difference. The search criteria used for mRNA datasets were normal tissue verses cancer tissue, and the cut-off fold change was 2. The *p*-value < 0.01 was considered significant (Goldman et al., 2020).

### GeneMania database

The GeneMANIA (Multiple Association Network Integration Algorithm) database is a computational approach for predicting protein function from functional association networks. We used the database to construct a gene interaction network by predicted interaction, physical interaction, co-expression, shared protein domains, and pathway analysis (http://genemania.org). The networks of MCMs were made to predict the most related genes as gene-associated networks. We have analyzed the gene functions of MCM10 as queried gene and constructed a network using the GeneMANIA database (Tang et al., 2017).

### Cell lines and culture conditions

Experimental Models: Cell lines C33 A (non-HPV), HeLa (HPV18) and SiHa (HPV16) were obtained from the National Centre for Cell Sciences (NCCS), Pune, India and cultured in Dulbecco’s Modified Eagle’s Medium (DMEM) supplemented with 10% FBS, 1% Glutamax and 1% penicillin streptomycin solution. HCK1T cells (normal cervical epithelium) were cultured in defined keratinocyte serum-free medium, supplemented with 5 ng/ml of epidermal growth factor and 50 μg/ml of bovine pituitary extract. The cells were maintained at 37°C with 5% CO_2_ in a humidified atmosphere.

### Immunofluorescence staining

Immunofluorescence staining was done according to (Nowińska and Dzięgiel, 2010). Anti-human MCM10, polyclonal antibody (Bethyl laboratories) was used at a dilution of 1:20. MCM10 stained images were captured in the EVOS 5000 Microscope (Thermo Fisher) and further analysed using ImageJ. The nuclear staining of each cell was measured and subtracted from the background fluorescence. Field selection was randomized.

### RNA extraction and quantitative RT-PCR (qRT-PCR)

Total RNA was isolated from cell lines using RNAiso Plus (Takara) according to the manufacturer’s protocol. Extracted RNA was quantified using the microvolume spectrometer (Colibri, Germany) and converted into cDNA using the PrimeScript RT Reagent Kit (Takara). Real-time PCR with TB Green Premix Ex TaqTM II (Takara) and qRT-PCR (BioRad)were used to quantify MCM10 and beta actin expression. Oligonucleotides used are as follows: CCA​GCT​CAC​CAT​GGA​TGA​T, GGT​CTC​AAA​CAT​GAT​CTG​GG, GGC​ATC​GGC​CAA​GAT​TCT​AC and GTC​TTC​CTC​CTC​ATC​CAT​GC for beta actin forward, beta actin reverse, MCM10 forward and MCM10 reverse respectively. To control the variability in mRNA expression levels, relative expression data was normalized to the mean of the housekeeping beta-actin gene.

### Statistical analysis

Experiments were performed in triplicate. The cervical cancer cell lines were compared against the normal cervical epithelial cell line. One-way ANOVA was used for the analysis, followed by Tukey’s multiple comparison. *p*-value < 0.05 was considered statistically significant and <0.01 was considered to be highly significant.

## Results

### Differential expression of MCMs in major cancers affecting women

**FIGURE 1:**
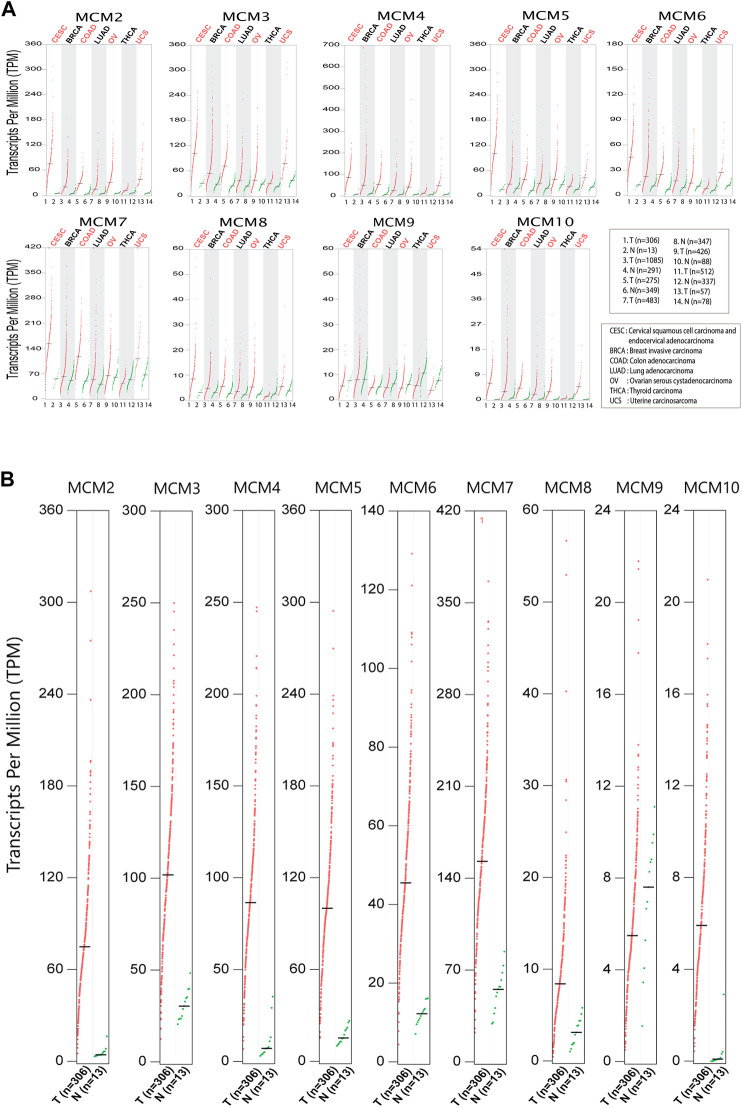
Differential expression of MCMs in cancer. **(A)** The expression of MCM2-10 in major cancers affecting women. The red dots represent the TPM value of each MCM in tumor tissues (T), while green dots represents the TPM value of each MCM in normal tissues (N). **(B)** Expression of MCMs in cervical cancer. The differential expression of MCM2-10 in cervical squamous cell carcinoma and endocervical adenocarcinoma (CESC). The red dots represent the TPM value of each MCM in tumor tissues, while the green dots represent the TPM value of each MCM in normal tissues.

In order to investigate the expression of the MCM proteins in the most common cancers that affect women worldwide, we looked into the biological databases dedicated to cancer data and oncogenomic research publicly available. We performed bioinformatic analysis to assess the expression and investigate the mutational status of MCM’s in cervical squamous cell carcinoma, endocervical adenocarcinoma (CESC) and six major cancers like breast invasive carcinoma (BRCA), colon adenocarcinoma (COAD), lung adenocarcinoma (LUAD), ovarian serous cystadenocarcinoma (OV), uterine carcinosarcoma (UCS) and thyroid carcinoma (THCA) that affect women worldwide. Analysis of the GEPIA datasets showed that, the mRNA expression levels of the MCMs were significantly increased in tumor tissues compared to normal (Figure 1A). We evaluated the gene expression profiling of MCMs in cervical cancer (Figure 1B) and found that all MCMs are overexpressed compared to normal. A significant expression pattern was obtained for MCM genes MCM2-10 in cervical squamous cell carcinoma and endocervical adenocarcinoma.

### Mutational status of MCMs in cervical cancer

**FIGURE 2:**
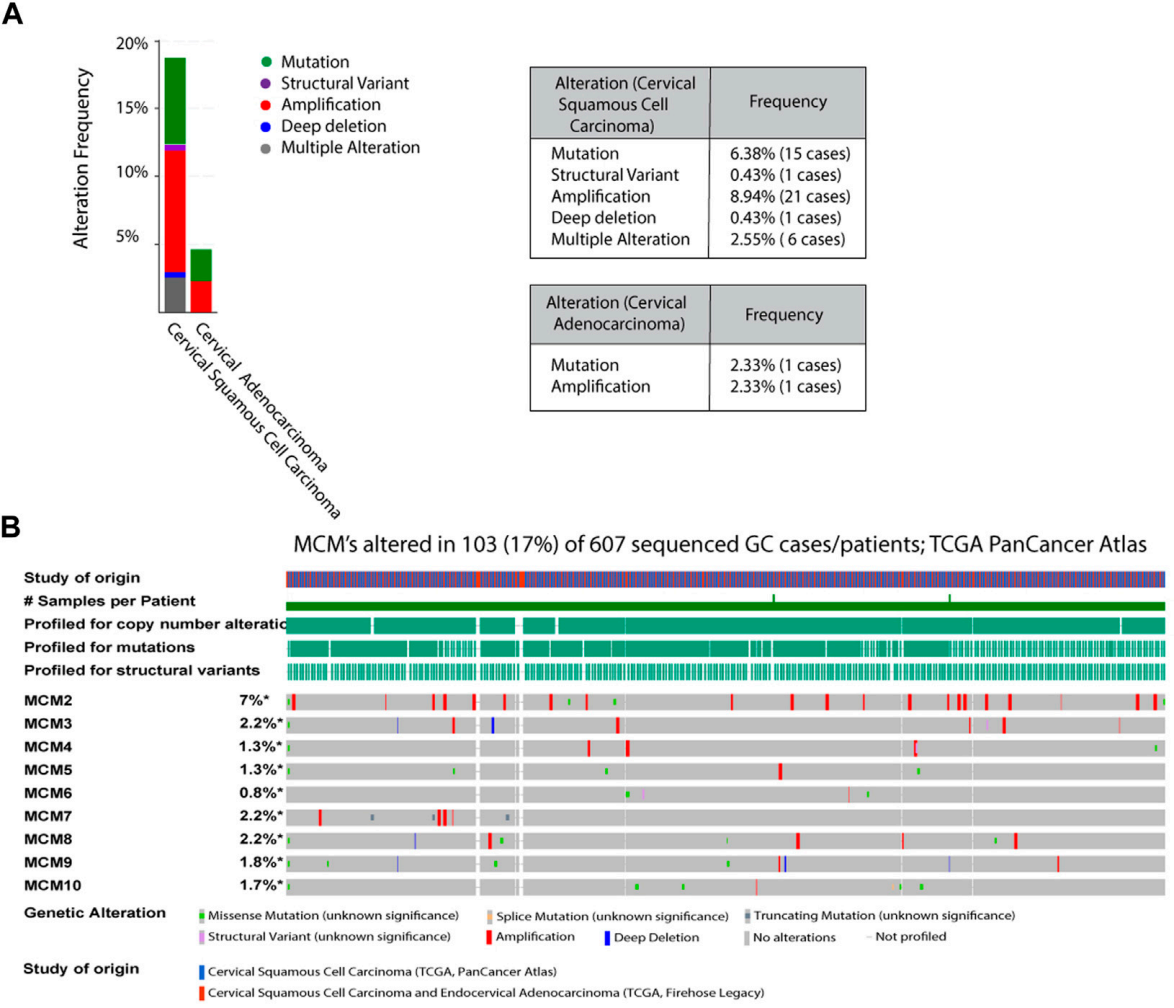
Analysis of MCM mutations in cervical cancer **(A)** The alteration frequencies of MCMs across cervical cancer studies as obtained from cBioPortal. Gene amplification denotes red bar, Mutation is represented as green bars, Structural variant as purple bars, Deep deletion is marked as blue bars, Multiple alteration as grey bars **(B)** Genetic Alterations: Amplification represented as red, deep deletion represented as blue, No alterations represented as grey.

To investigate the aberrant expression of MCMs in cervical cancer, we analyzed the genomic alterations of each MCM gene using cBioPortal. The alteration frequency of selected genes is shown in Figure 2. The queried genes are altered in 18.72% of 235 cases of cervical squamous cell carcinoma and 4.65% of 43 cases of cervical adenocarcinoma. The amplification frequencies of cervical squamous cell carcinoma and cervical adenocarcinoma were 8.94% and 2.33% respectively, while mutation frequencies were 6.38% and 2.33% (Figure 2A). In a 607-patient TCGA cohort, 103 (17%) of the 607 patients showed genetic changes. Alterations were seen in 7% of the clinical cases associated with MCM2, while MCM3, MCM7, and MCM8 had 2.2% of the alterations each. Various types of alterations, like missense mutation, splice mutation, truncations, and gene amplification, are prominent molecular events associated with the MCM gene in the case of cervical cancer (Figure 2B). MCM2 had the most amplification events, and MCM10 had more missense mutations. Amplification and missense mutations were the most common genetic changes among MCMs. Such mutational features again confirm the dysregulated phenotype shown in different cancer types.

### MCM10 is significantly overexpressed in cervical cancer

**FIGURE 3:**
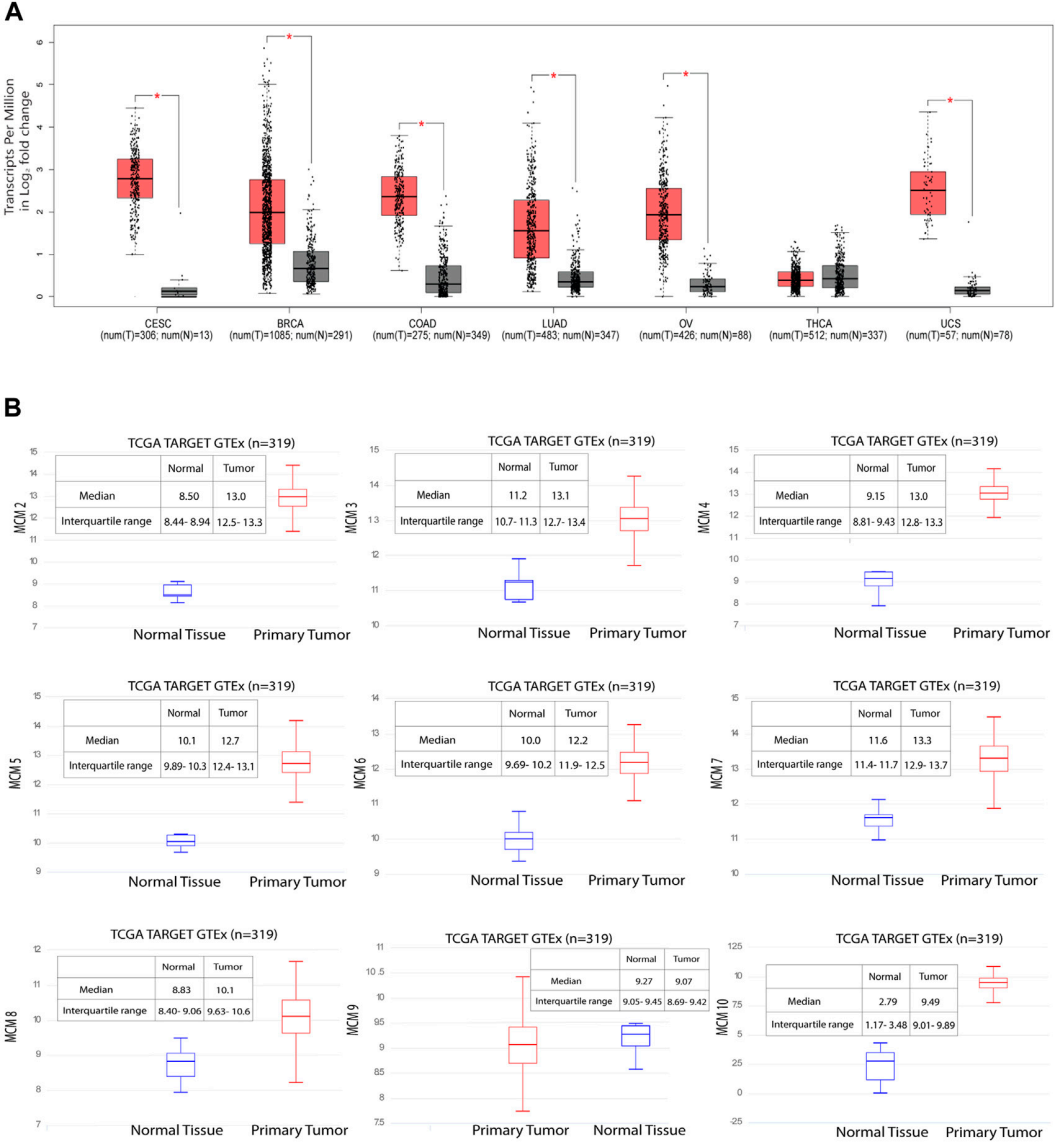
Significant overexpression of MCM10 in cancer. **(A)** MCM10 expression in different cancers. Box and whisker plot based on GEPIA database, depicts the expression levels of MCM10 in CESC, BRCA, COAD, LUAD, OV, THCA and UCS. T: Tumor (Red), N: Normal (Grey). **(B)** Gene expression data of 319 samples available from the UCSC Xena database. The *Y* axis shows the RNAseq- RSEM norm_count Unit: log2 (norm_count+1) gene expression in cervical cancer for MCM2-7, MCM8, MCM9 and MCM10. By comparing the median of normal and tumor, the expression level of MCMs fold change was obtained. Blue box plot represents normal tissue whereas red box plot represents tumor tissues.

MCM10 is a key molecule that promotes initiation of DNA replication by origin unwinding and we wanted to investigate the expression of MCM10 in cancers. We observed a 0.978–3.401 fold change in the expression levels of the MCM proteins (Supplementary Table S1), from patient samples emphasizing the fact that these proteins can be potential biomarkers, taking into account both specificity and sensitivity. Using the GEPIA database, we investigated MCM10 expression of the top six cancers that affect women, including cervical cancer. Our analysis revealed that the transcriptional level of MCM10 expression is significantly higher in cervical cancer (CESC) than in any other cancer types like BRCA, COAD, LUAD, OV, UCS and THCA (Figure 3A). To further explore the genetic interaction networks for the MCM complex, we performed data mining and constructed an interaction network using GeneMANIA software (Supplementary Figure S1). We explored the genetic interaction network of MCM10 in humans, which is one of the significantly overexpressed proteins. It is shown to interact with other genes of the replication complex such as MCM2-7, CDT1, ORCs, DBF4, CDCs, RPA4, POLE2, ATRIP and SIRT1 which reinstates its importance in the regulation of replication initiation. MCM10 also relates to other traits like physical interaction (77.64%), co-expression (8.01%), predicted interaction (5.37%), shared protein domains (0.60%), and similarity in pathway (1.88%) (Supplementary Figure S1A–F). This data shows how MCM10 expression is important in cervical cancer. We further analyzed the transcriptional level of the MCMs in cervical cancer tissues and compared it to that in normal cervical tissues based on the data available in UCSC Xena. Among all the MCMs, the RNA levels of MCM10 in the tumor showed a 3.4-fold increase compared to the control (Figure 3B, Supplementary Table S1). MCM10 is thus a promising candidate for early detection of cervical cancer.

### MCM10 overexpression is associated with aggressive cervical cancer cell lines

**FIGURE 4:**
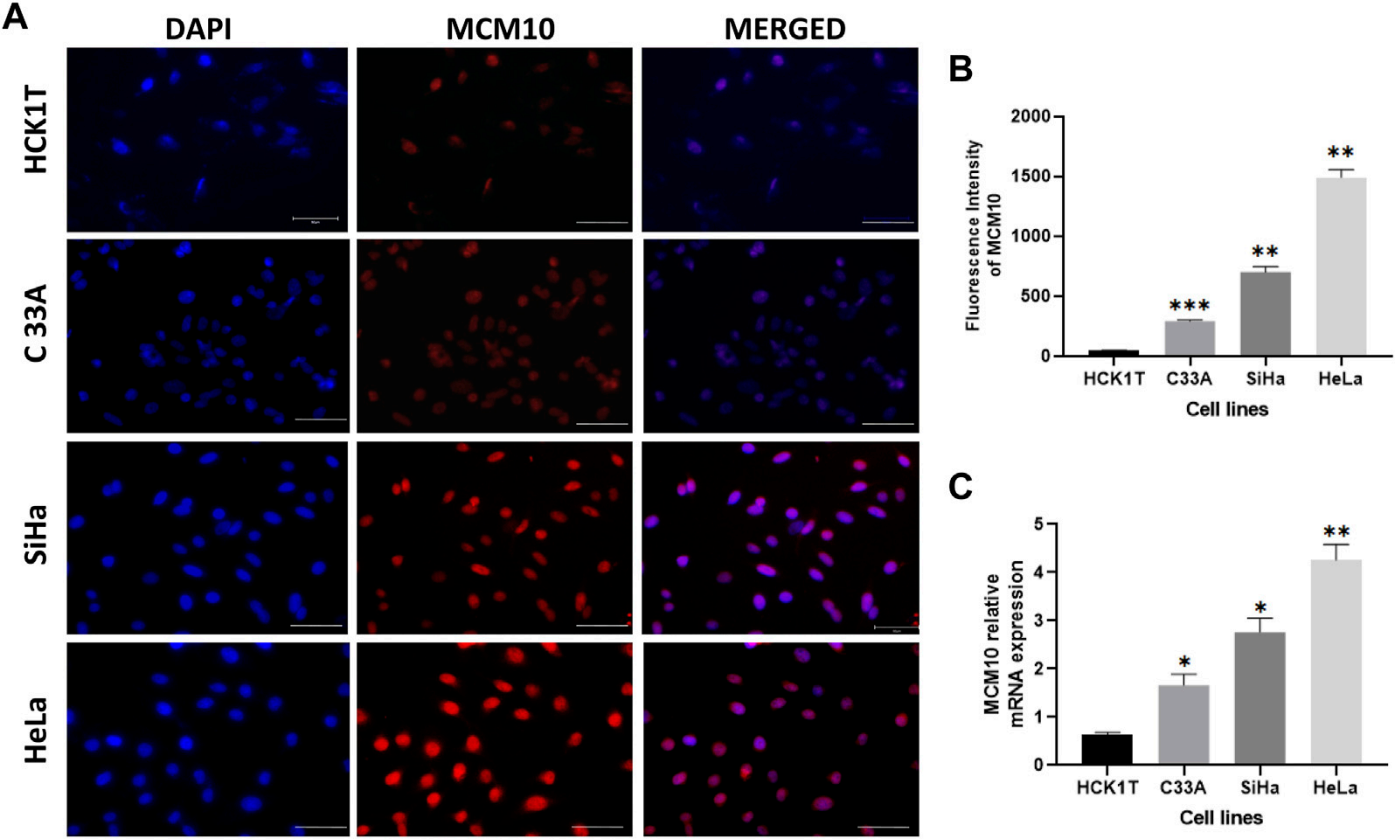
MCM10 expression in cervical cell lines (HCK1T, C33A, SiHa, and HeLa). **(A)** Immunofluorescence staining of DAPI (blue), MCM10 (red) and merged image (purple) shows the difference in the intensity of staining (Magnification ×40, Scale bars 50 m). **(B)** Quantification of immunofluorescence using ImageJ software. Error bar shows the standard deviation. **(C)** Relative mRNA expression of MCM10 in normal cervical epithelium cell line (HCK1T) and the human cervical cancer cell lines (C33A, SiHa, and HeLa). Error bar shows the standard deviation.

In order to understand if MCM10 expression is associated with the aggressiveness of cervical cancer, we looked into the mRNA expression pattern of MCM10 in four cervical cell lines HeLa (HPV18), SiHa (HPV16), C33A (non-HPV) and HCK1T (normal cervical epithelium). We first looked into the protein levels to understand if MCM10 expression correlated with the aggressiveness of cancer. Our data indicates that increased MCM10 protein levels indeed correspond with the highly aggressive HPV variant of cervical cancer (Figure 4). Staining showed that the MCM10 protein was localized to the nucleus of the cervical cancer cell lines. There was intense staining in the HeLa (HPV18), whereas reduced intensity was noted in SiHa (HPV 16), with further reduced signal for C33A (non-HPV) (Figure 4A). The quantitation of the fluorescence intensity indicates that MCM10 intensity increases with the aggressiveness of cancer (Figure 4B). We further wanted to see if the protein expression correlates with the mRNA expression levels, and our qRT-PCR data confirms that MCM10 expression indeed corresponds with the aggressive cancer phenotype (Figure 4C). The relative expression of MCM10 were HeLa 4.027 ± 0.321, SiHa 2.928 ± 0.288, C33 A 1.526 ± 0.23, and HCK1T 0.607 ± 0.041 as shown in Figure 4C. These results indicate a strong association between increased MCM10 expression and the aggressiveness of cervical cancer.

## Discussion

Promising cancer biomarkers, such as the DNA replication proteins, required for precise genome duplication, are overexpressed in almost all types of cancer (Ryu and Driever, 2006; Yim and Park, 2006; Saritha et al., 2018). Uncontrolled DNA replication is one of the major characteristics of dysplasia and malignant cells (Yu et al., 2020; Fu et al., 2021). Both overexpression and inhibition of the minichromosome maintenance proteins are significantly linked to tumor formation (Cheng et al., 2020; Lei et al., 2021) and have been proposed as potential proliferation markers. Apart from their potential utility as biomarkers, they can also be used for therapy to regulate the process of genome duplication (Ryu et al., 2005). Because of their higher sensitivity and specificity in context to diagnostics, MCMs have an advantage over traditional cell cycle markers like Ki67 and PCNA (Vargas et al., 2008). Most of the MCM positive cells in cervical cancer are distributed at the surface of the malignant and premalignant epithelial cells, which can be detected and confirmed with immunostaining results (Jackson et al., 2014). In this study, we have systematically analysed the expression of MCM proteins, which are involved in origin licencing and DNA unwinding in six major malignancies that affect women, with emphasis on cervical cancer (CESC). We mined the publicly available biological databases dedicated to cancer data and investigated the transcriptional abundance of the different MCM subunits. Based on data available at TCGA, cBioPortal, UCSC Xena, and GEPIA, our analysis shows that, like in other cancer types, the mRNA transcripts of MCMs are elevated in the tumour tissue of cervical cancer patients (Figure 1).

A common cause of genomic instability is the defect in DNA damage response and inaccurate DNA repair that leads to chromosomal alterations. This involves chromosome number and structural changes (mutations, including deletions and amplification events) leading to chromosomal instability in cancer cells (Rhind, 2006; Abbas et al., 2013). The altered frequencies of mutations in adenocarcinoma and squamous cell carcinoma in cervical cancer patients are hallmark features of the disease (Negrini et al., 2010) (Figure 2A). Among 298 cases, 19% had an alteration in the MCM gene (Figure 2B). We found that the MCM2 gene had the most structural alterations, whereas the MCM10 gene carried a number of missense mutations but lacked amplification events and deep deletions. MCM10 is one of the key regulators of DNA replication initiation and is also essential for maintaining genome integrity (Baxley and Bielinsky, 2017). Interestingly, though not a member of the core MCM helicase complex, it triggers replication origin firing and initiates unwinding along with the GINS complex and Cdc45 (Yeeles et al., 2015; Lõoke et al., 2017; Mayle et al., 2019; Rios-Morales et al., 2019). This crucial event is disrupted in the case of cancer cells (Das et al., 2013; Douglas and Diffley, 2016). Cdt1 and Cdc6 overexpression along with defects in the p53 pathway is known to be correlated with the aggressiveness of cancer, EMT transition, cancer invasion, and metastasis (Liontos et al., 2007), a conditions we observe for MCM10 expression in cervical cancer cell lines. Here we report the expression of MCM subunits, in particular MCM10, which was found to be significantly upregulated in aggressive forms of cervical cancer (Figure 3), and we speculate that MCM10 levels could be an important marker for the aggressiveness of cancer.

In one of our earlier studies, we showed that MCMs are distributed heterogeneously across the genome, and that replication origins with more MCMs bound have a higher probability of firing early in the S-phase of the cell cycle (Rhind, 2006; Das et al., 2015). In this scenario, molecules like MCM10 are crucial for all those licenced origins to have a higher number of the helicase MCM2-7 bound to actually fire early in S-phase. In fact, the number of activated origins in cancer cells, is statistically larger than in normal cells (Valenzuela et al., 2011), which suggests that origin usage flexibility increases in cancer cells and molecules like MCM10 are needed to activate additional origins of replication. Measuring the transcript levels of these limiting proteins can thus act as important biomarkers to assess the aggressiveness of cancer. Activation of inefficient/dormant origins as a result of replicative stress becomes important to counter the high frequency of stalled replication forks (Ge et al., 2007). MCM10 plays a crucial role in activating such origins. When we investigated the mRNA expression profile for MCM10, it was found to be high in CESC when compared to BRCA, COAD, LUSC, OV, THCA, and UCS (Figure 3A). Though the transcript level of MCM10 is lower compared to the other MCMs, the relative fold change is robust (3.4-fold in the case of CESC) when compared to the control samples, and thus MCM10 is a promising biomarker compared to other MCMs (Figure 3B).

Based on our experimental and *in silico* findings, we propose MCM10 levels are significantly upregulated in aggressive forms of cervical cancer as evident from the overexpression signal obtained from cervical cancer cell lines. One limitation of this study is the supporting data from patient samples for the correlation of MCM10 expression and the different stages of cervical cancer. The expression of the DNA replication protein MCM10, as seen in more aggressive cervical cancers such as Hela cells (Sanchez-Sandoval and Gomora, 2019), results in the initiation of additional DNA replication origins. Increased MCM10 fluorescence and mRNA transcript signal compared to normal cells support this (Figure 4 A, B). One interesting observation showed that decreasing the levels of MCM10 led to decreased growth in cancer cells, whereas normal cells were not affected (Murayama et al., 2021). This shows that MCM10 acts as a limiting factor for dealing with origin activation in cancer cells. Thus, the level of MCM10 plays a crucial role in compensating for DNA replication stress in cancer cells and facilitates genome duplication in S-phase by firing additional origins that are primed and licensed to fire. MCM10 can play a key role in the detection of early stages of cervical cancer and screening younger women for MCM10 expression levels can aid in preventing cervical cancer from progressing to its advanced stages. Hence, we propose MCM10 as an important marker to assess cervical cancer progression.

## Data Availability

The datasets presented in this study can be found in online repositories. The names of the repository/repositories and accession number(s) can be found in the article/Supplementary Material.

## Abbreviations

HPV, Human papillomavirus; HCK1T, Human Cervical Keratinocyte; BRCA, Breast invasive carcinoma; CDT1, Chromatin licensing and DNA replication factor 1; CDC, Cell division cycle; CESC, Cervical squamous cell carcinoma and endocervical adenocarcinoma; CMG, Cdc45/Mcm2–7/GINS; COAD, Colon adenocarcinoma; EMT, Epithelial-to-Mesenchymal Transition; GEPIA, Gene Expression Profiling Interactive Analysis; GTEx, Genotype-Tissue Expression; LUAD, Lung adenocarcinoma; MCM, Minichromosome Maintenance Protein; NCCS, National Centre for Cell Science; ORC, Origin Recognition Complex; OV,Ovarian serous cystadenocarcinoma; UCS, Uterine Carcinosarcoma; UCSC Xena, University of California Santa Cruz Xena; TCGA, The Cancer Genome Atlas; THCA, Thyroid carcinoma; TPM, Transcript per million.
