# Supplementary material for: MCM10 expression is linked to cervical cancer aggressiveness
Source: Front Mol Med. 2023 Feb 22;3:1009903. doi: 10.3389/fmmed.2023.1009903 (PMC11285692; doi:10.3389/fmmed.2023.1009903)
Supplement: Supplementary file 1 [file Table1.docx]

**Supplementary Table: Significant changes in MCM expression at the transcription level between cervical tumor and normal tissues using UCSC Xena Database.**

| Cancer  types | Gene Symbol | Median (Tumor) | Median  (Normal) | (Fold Change) |
| --- | --- | --- | --- | --- |
| CESC | MCM2 | 13.0 | 8.50 | 1.529 |
|  | MCM3 | 13.1 | 11.2 | 1.169 |
|  | MCM4 | 13.0 | 9.15 | 1.420 |
|  | MCM5 | 12.7 | 10.1 | 1.257 |
|  | MCM6 | 12.2 | 10.0 | 1.220 |
|  | MCM7 | 13.3 | 11.6 | 1.146 |
|  | MCM8 | 10.1 | 8.83 | 1.143 |
|  | MCM9 | 9.07 | 9.27 | 0.978 |
|  | MCM10 | 9.49 | 2.79 | 3.401 |
