# Supplementary figures and images for: MCM10 expression is linked to cervical cancer aggressiveness
Source: Front Mol Med. 2023 Feb 22;3:1009903. doi: 10.3389/fmmed.2023.1009903 (PMC11285692; doi:10.3389/fmmed.2023.1009903)

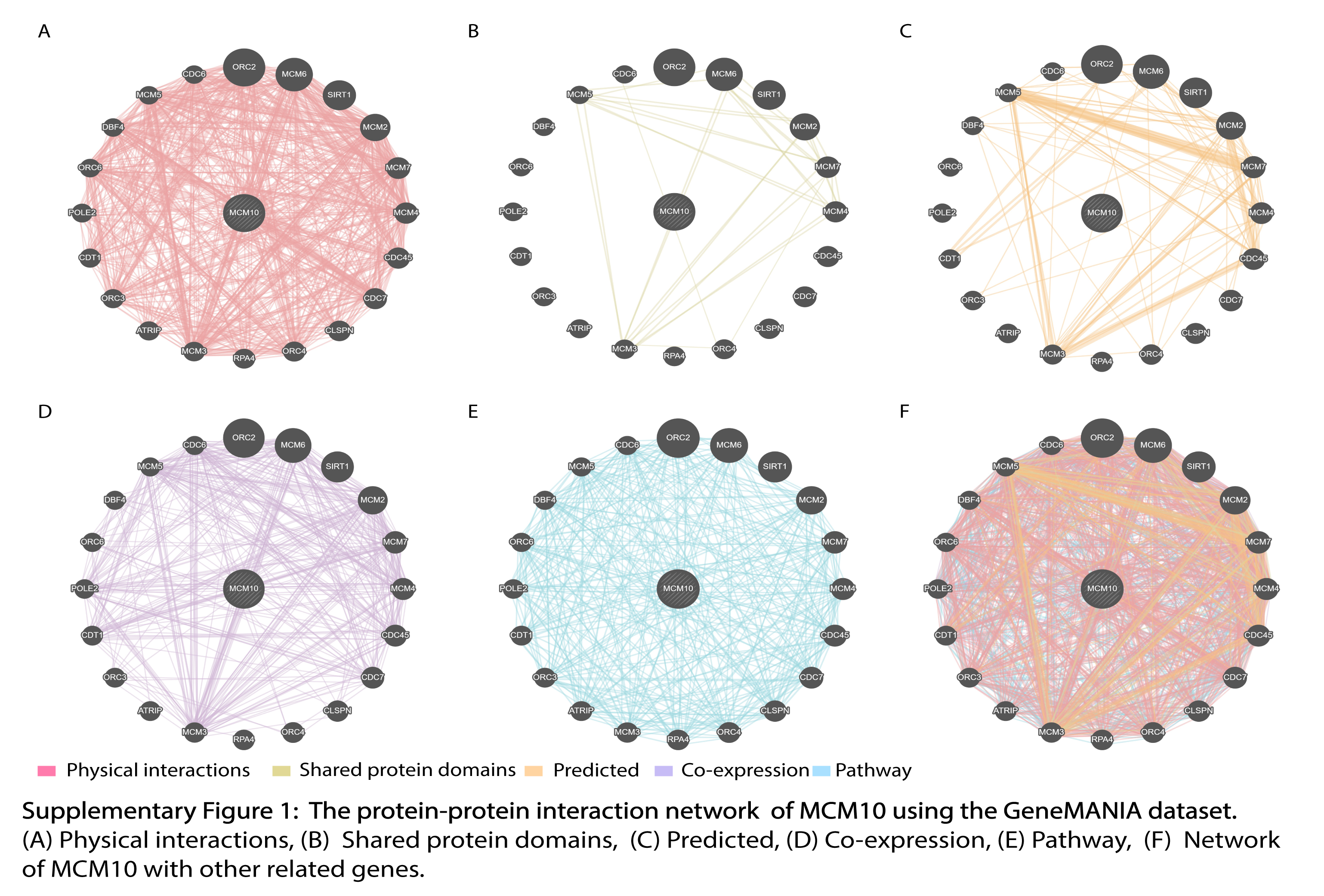

Supplement: Supplementary file 2 [file Image1.JPEG]
